# The intersection of disorders of sex development and cardiovascular diseases

**DOI:** 10.1186/s13293-025-00814-4

**Published:** 2026-03-05

**Authors:** Satwat Hashmi, Rédouane Aherrahrou

**Affiliations:** 1 Department of Biological and Biomedical Sciences, Aga Khan University, Karachi, Pakistan; 2 Institute for Cardiogenetics, Universität zu Lübeck, DZHK (German Centre for Cardiovascular Research), Partner Site Hamburg/Kiel/Lübeck, Marie-Curie-Str. Haus 67/BMF, Lübeck, 23562 Germany; 3 University Heart Centre Lübeck, Lübeck, 23562 Germany; 4 A.I. Virtanen Institute for Molecular Sciences, University of Eastern Finland, Joensuu and Kuopio, Finland; 5 Immunology Center of Georgia, Medical College of Georgia at Augusta University, Augusta, United States of America; 6 Department of Pharmacology & Toxicology, Medical College of Georgia at Augusta University, Augusta, United States of America

## Abstract

**Background:**

Disorders of Sex Development (DSD) refer to a group of congenital conditions where chromosomal, gonadal, or anatomical sex development is atypical. Cardiovascular diseases (CVD) are a leading cause of illness and death worldwide, often resulting in serious conditions like heart attacks, strokes, and heart failure. Recent research suggests that shared mechanisms may link DSD and CVD. This study aims to investigate the shared genetic mechanisms between DSD and CVD, which could uncover common biological pathways involved in their development.

**Methods:**

We performed a comprehensive analysis using a dataset of 169 genes associated with 46XY DSD and corresponding genes linked to CVD, gathered from published research. The overlapping genes between them were identified and grouped into four biological processes: transcription factors, signaling pathways, hormonal regulation, and developmental regulation.

**Results:**

In this review, we explored the potential link between recognized 46XY DSD genes and CVD. We found 25 genes that are shared between the 46 XY DSD and CVD, suggesting a genetic connection between the two conditions. These shared genes fall into categories such as transcription factors, signaling pathways, hormonal regulation, and developmental regulation. This gives us valuable insights into how these genetic factors might affect cardiovascular health in people with DSD. Each gene and its role in 46XY DSD and CVD will be discussed separately. We will also address challenges and provide suggestions for a better understanding of the genetics involved. Additionally, the review will outline future research directions crucial for advancing our understanding of the connection between 46XY DSD and CVD, with the goal of improving health outcomes for affected individuals.

**Conclusions:**

Our findings suggest a genetic link between 46 XY DSD and CVD, indicating that shared molecular mechanisms may play a role in the development of both conditions. These insights into the connections could have important implications for personalized medicine, potentially allowing for treatments that target both 46 XY DSD and CVD.

**Supplementary Information:**

The online version contains supplementary material available at 10.1186/s13293-025-00814-4.

## Abstract (plain language summary)

Disorders of sex development (DSD) are conditions that affect how a person’s body develops in terms of sex characteristics. People with DSD may have chromosomes, hormones, or physical features that differ from typical male or female patterns. Cardiovascular disease (CVD), which includes conditions like heart attacks and strokes, is the leading cause of death worldwide. While DSD and CVD are very different, recent research suggests they may be connected through shared genetic factors.

In this article, we explore the possibility that certain genes involved in the development of 46 XY DSD and CVD might overlap. This overlap could mean that people with 46 XY DSD are at an increased risk for heart disease due to these shared biological pathways. Identifying these connections may help scientists and doctors develop more personalized treatments for both 46 XY DSD and CVD, potentially improving health outcomes for individuals with 46 XY DSD who are at risk of cardiovascular issues.

This research highlights the importance of exploring connections between seemingly unrelated conditions. Discovering these links can lead to better diagnosis, treatment, and prevention efforts. Our findings may also open opportunities for further studies that address the specific cardiovascular health risks faced by people with 46 XY DSD.

**Supplementary Information:**

The online version contains supplementary material available at 10.1186/s13293-025-00814-4.

## Highlights

We identified 25 genes shared between disorders of sex development (46 XY DSD) and cardiovascular diseases (CVD), suggesting a significant genetic connection between these conditions. These genes were categorized into key biological processes, including transcription factors and signaling pathways, both of which are central to the development of 46 XY DSD and CVD. This overlap highlights potential shared mechanisms underlying these distinct conditions and provides new insights into how 46 XY DSD may influence cardiovascular health, with important implications for clinical practice. Further investigation into these genetic pathways is essential, as a more detailed understanding could enable the development of targeted and effective treatments for individuals affected by both 46 XY DSD and CVD.

**Supplementary Information:**

The online version contains supplementary material available at 10.1186/s13293-025-00814-4.

## Introduction

Disorders of Sex Development (DSD) encompasses a diverse array of congenital conditions characterized by atypical chromosomal, gonadal, or anatomical sex development [1]. These conditions can influence sexual differentiation and function, manifesting in a wide range of phenotypes, from atypical genitalia to complete sex reversal. Characteristics such as ambiguous genitalia, gonadal dysgenesis, hormonal imbalances, chromosomal variations, infertility, and atypical puberty are commonly observed in individuals with DSD [2]. The etiologies of DSD are multifaceted, often involving mutations or dysregulation in genes pivotal for steroidogenesis, hormone synthesis, and other critical developmental pathways [3].

Cardiovascular Diseases (CVD), on the other hand, remains the leading cause of mortality in men and women worldwide [4]. This can result in severe clinical outcomes such as myocardial infarction (MI), stroke, and heart failure [5–7]. The pathogenesis of CVD is complex and multifactorial, involving an interplay of genetic predispositions, lifestyle factors (including diet, physical activity, and smoking), and comorbid medical conditions such as hypertension, diabetes, and hyperlipidemia [5, 8].

**Fig. 1:**
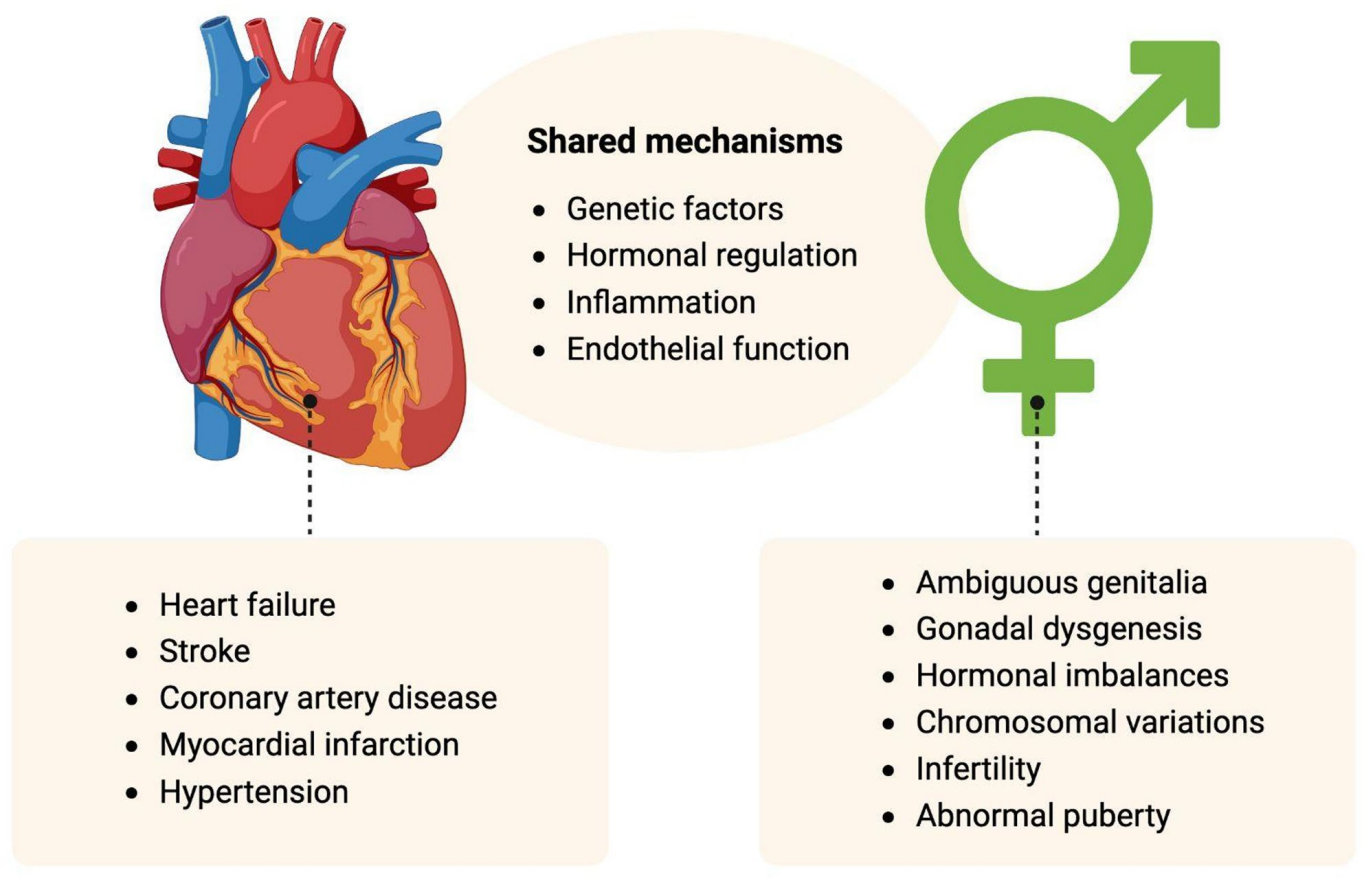
Shared pathophysiological mechanisms and associated conditions between cardiovascular diseases (CVD) and Disorders of Sex Development (DSD)

Despite the apparent differences between DSD and CVD, emerging research suggests intriguing genetic and pathophysiological connections between these conditions. Shared mechanisms between DSD and CVD, such as genetic factors, hormonal regulation, inflammation, and endothelial dysfunction, suggest that these pathways may overlap (Fig. 1).

An example of this is a recent study by Lucas-Herald et al. (2024) which showed that 20% of young individuals with 46XY DSD had hypertension in childhood, as compared to 5% of the global prevalence in children [9]. Another study reported an association between 46 XY DSD and congenital heart disease. In this study, a pathogenic variant of *GATA4*, c.487 C > T(p.Pro163Ser), identified through whole-exome sequencing, was associated with atrial septal defect in 46XY DSD individuals [10].

**Fig. 2:**
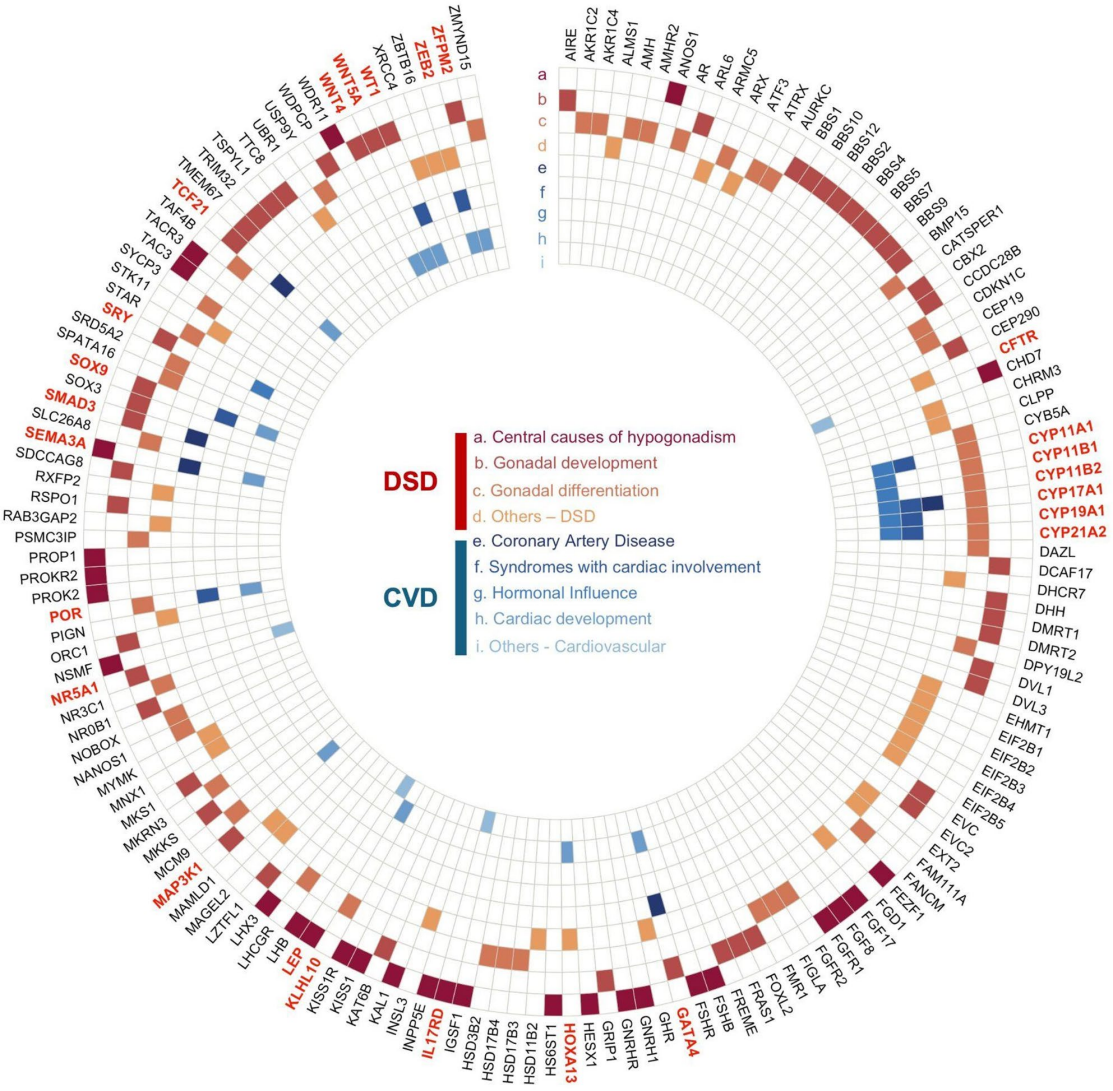
Shared Genetic Factors Between 46 XY DSD and CVD. This circular plot illustrates the genetic intersections between 46 XY DSD and CVD, highlighting specific genes associated with various aspects of these conditions. Divided into sections based on genetic influence, each category is color-coded: red shades (**a**-**d**) for 46 XY DSD-related categories and blue shades (**e**-**i**) for cardiovascular-related categories

Figure 2 shows the shared genes between 46 XY DSD and CVD, about 25 out of 169, as highlighted in bold red, which supports the idea that genetic factors may drive the overlapping molecular mechanisms between the two conditions.

**Fig. 3:**
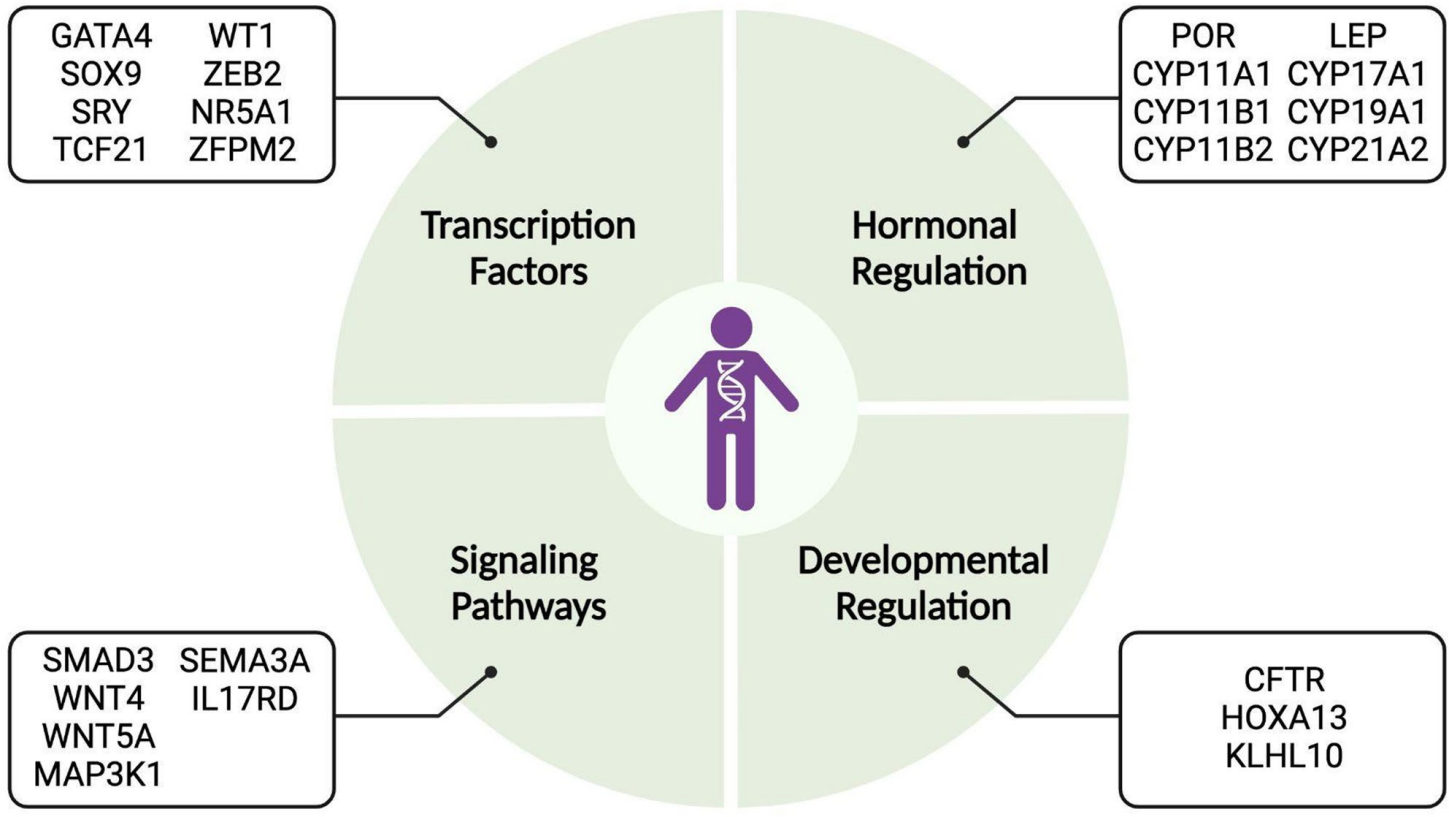
25 shared genes associated with both CVD and 46 XY DSD. The 25 shared genes are categorized into four different groups: transcription factors, signaling pathways, hormonal regulation, and developmental regulation

This review will explore the genetic intersections between 46XY DSD and CVD in detail (Fig. 3), examining how shared genetic factors and mechanisms may contribute to both conditions. It will also discuss challenges and provide further suggestions for understanding the genetics involved. Finally, the review will highlight future research directions essential for advancing our understanding of the connection between DSD and CVD, ultimately aiming to improve health outcomes for affected individuals.

## Shared genes between DSD and CVD

In this study, we selected 169 genes associated with 46 XY DSD [11, 12] and searched for any associations with CVD by reviewing related studies (PubMed, Google Scholar) to identify genetic overlaps between these conditions. A total of 25 DSD 46XY genes were identified as being associated with CVD. The DSD genes that showed an evident link to CVD, either through animal or human studies, are displayed in the Supplementary Tables 1 and 2.

These overlapping genes were grouped into four categories: transcription factors, signaling pathways, hormonal regulation, and developmental regulation. This classification provides insight into the diverse biological processes that may underlie the genetic intersection of these conditions. Figure 3 illustrates the shared genes, with further details on each gene and its role in DSD and CVD, which will be discussed in the subsequent sections.

## Transcription factors

### GATA4 (GATA binding protein 4)

*GATA4* is vital for the early development of several organs, including the testis [13]. GATA4 works with cofactors, such as NR5A1 and ZFPM2, to modulate gene expression of *SOX9* and *AMH*, which are involved in sex determination and differentiation [14, 15]. XY mouse embryos with a homozygous *Gata4* knock-in allele prevent *Gata4* from binding to *fog2* and halt the differentiation of testis [16]. Additionally, GATA4 plays a crucial role in cardiomyocyte hypertrophy [17]. Overexpressing GATA4 in cardiomyocytes via recombinant adenovirus induces significant hypertrophy [18]. Moreover, there is a progressive increase in heart-to-body weight ratio, development of cardiomyopathy, and activation of hypertrophy-related genes in transgenic mice with 2.5-fold higher *Gata4* expression, confirming GATA4 as a key regulator of hypertrophy both *in vitro* and *in vivo* [18]. Furthermore, Muiya et al. (2013) suggest that GATA4 could be a risk factor for congenital heart disease (CHD), coronary artery disease (CAD), and MI, each independently, and also serves as a metabolic risk trait for CVD [19]. The several causative haplotypes for MI and CAD, which include the 3′UTR of the *GATA4* gene, emphasize that this locus plays crucial roles in the development of diseases such as CAD and MI [19].

### SOX9 (SRY-box transcription factor 9)

*SOX9* plays a pivotal role in regulating chondrogenesis [20], sex differentiation and testis differentiation [21]. Early deletion of *SOX9* in the gonads caused a complete male-to-female sex reversal in XY individuals [22]. In addition, heterozygous loss-of-function mutations in *SOX9* are linked to Campomelic Dysplasia, a severe skeletal malformation syndrome that causes 70% of affected XY individuals to exhibit female development. It is also linked to XY sex reversal in humans [23–25]. SOX9 also plays a significant role in the pathogenesis of various fibrotic diseases, such as liver fibrosis, glomerulosclerosis, and heart valve calcification [26–28]. Scharf et al. (2019) found that *Sox9* is elevated in scar tissue after MI in mice, indicating its involvement in tissue repair [29]. Deleting *Sox9* in fibroblasts improved left ventricular function, reduced scar formation and leukocyte infiltration, and lowered gene expression levels related to inflammation, the extracellular matrix, and proteolysis [29]. Additionally, *Sox9* in cardiomyocytes specifically influences hypertrophy and early fibrosis following cardiac pressure overload, and loss of *Sox9* in cardiomyocytes has been found to postpone the onset of cardiac hypertrophy and fibrosis [29].

### SRY (sex-determining region Y)

The discovery of the *SRY* gene on the Y chromosome has been a groundbreaking milestone in our understanding of sex determination and the processes driving male and female development [30]. *SRY* encodes a transcription factor that binds to and activates the testis-specific enhancer of the related gene *SOX9* [31]. Further studies confirmed its essential role in testicular development [32, 33]. Cai et al. (2015), showed that the *SRY* gene transferred by extracellular vesicles (EVs) accelerates atherosclerosis by promoting leukocyte adherence to endothelial cells, highlighting its role in inflammatory response [34]. EVs increase the expression level of SRY protein in immune cells and endothelial cells in male patients with CAD. In addition, injecting SRY-EVs into mice accelerates atherosclerosis, highlighting its role in CAD [34].

### TCF21 (transcription factor 21)

*TCF21*, which is the basic helix-loop-helix (*bHLH*) gene, is one of the direct downstream targets of *SRY* gene [35]. Bhandari et al. (2012) identified the downstream targets of *TCF21* and the potential cascade of *bHLH* genes that support Sertoli cell differentiation. This cascade, involving SRY followed by TCF21 and then SCX, appears to play a role in Sertoli cell fate determination and consequent differentiation [36]. Also, it has been demonstrated that TCF21 plays a crucial role in cell differentiation, particularly in organ development [37]. Regarding heart development, retinoic acid signaling also regulates TCF21, which in turn inhibits the differentiation of epicardium-derived cells into smooth muscle cells. These cells are vital for forming the cardiac fibrous matrix and coronary vasculature, which are essential for maintaining myocardial function and integrity [38]. Furthermore, SNPs related to *TCF21* have been closely linked to CAD. The study found that polymorphism disrupts the binding of miRNA to *TCF21* [39]. It is also associated with an increased risk of major adverse cardiovascular events in patients with CAD [40, 41]. Additionally, these SNPs (rs12190287, rs12413409, rs1412444, rs1746048 and rs4977574) have been significantly linked to MI in the Chinese population [42]. Moreover, in rs12190287, the change from G to C has been identified as a susceptibility locus for hypertension, with the C allele contributing to an increased risk for hypertension [43].

### WT1 (WT1 transcription factor)

*WT1* is essential for male development. A heterozygous loss of this gene in patients with WAGR syndrome results in hypospadias and cryptorchidism [44]. *Wt1* knockout mice showed defects in mesothelia, lungs, and heart along with the absence of gonadal development, and they typically die around day 14 of gestation [45]. Furthermore, Klamt et al. (1998) reported that Frasier syndrome, which is characterized by kidney failure, and complete gonadal dysgenesis, is likely triggered by specific intronic point mutations in the *WT1* gene, especially those affecting a CpG dinucleotide [46]. Beyond that, Velecela et al. (2013) found that WT1 helps control the levels of two chemokines (Ccl5 and Cxcl10) in cells around the heart [47]. As WT1 decreases, these chemokines increase, which can affect cell movement and growth. WT1 also helps reactivate these cells when the heart is damaged [47]. Díaz del Moral et al. (2021) reported that *WT1* is expressed at low levels in 20–25% of embryonic cardiomyocytes [48]. The conditional decrease of *WT1* led to atypical development of the sinus venosus and atrium, a lack of pectinate muscles, a thin ventricular myocardium, and in some cases, defects in the interventricular septum and cardiac wall, as well as ventricular diverticula and aneurysms [48]. Moreover, adult *Wt1* mutant mice exhibited electrocardiographic anomalies, indicating that WT1 in the myocardium is essential for normal cardiac function [48].

### ZEB2 (zinc finger E-box binding homeobox 2)

*ZEB2* gene, also called SIP1 (Smad-interacting protein 1), mutations cause Mowat–Wilson syndrome (MWS) through de novo heterozygous mutations [49, 50]. MWS is characterized by multiple congenital anomalies, like microcephaly, agenesis of the corpus callosum, conotruncal heart defects, urogenital malformations, Hirschsprung disease, and hypospadias. They also have severe intellectual disability, epilepsy and distinctive facial appearance [51, 52]. In CVDs, several CAD risk variants were found near the *ZEB2* gene. These variants likely work together in the atherosclerotic arterial wall and adipose tissues by influencing metabolic and lipid functions [53]. *ZEB2* also interacts with *SMAD3*, another CAD-associated gene, suggesting they may work together in regulating the processes that contribute to atherosclerosis and vascular remodeling [54]. Furthermore, when *Zeb2* is lost in vascular smooth muscle cells (VSMCs) in mice, these cells struggle to switch off their contractile function and change into a fibroblast-like state. Instead, they begin to form chondrocytes, which reflect features seen in high-risk atherosclerotic plaques in human coronary arteries. As a result, dysfunction of ZEB2 may be associated with the development and progression of CVDs [55].

### NR5A1 (nuclear receptor subfamily 5 group A member 1)

*NR5A1* is essential for regulating steroid production and reproductive development. It plays a key role in the formation of adrenal glands and gonads [56]. Specifically, *NR5A1* is crucial for testicular differentiation and the expression of the anti-Müllerian hormone (*AMH*) gene in Sertoli cells [57, 58]. It also helps synthesize enzymes that produce steroids in Leydig cells during the development of 46 XY embryos, which influences testosterone production and genital formation [59, 60]. Mutations in the *NR5A1* gene are closely linked to DSD and fertility issues [61]. The study revealed that NR5A1 levels decrease in response to signals like angiotensin II, potassium chloride, and cyclic adenosine monophosphate (cAMP), which suggests that these substances reduce NR5A1 activity. This reduction is significant because NR5A1 is crucial for regulating aldosterone production [62]. The research also showed that *NR5A1* mRNA is more abundant in adrenal tissues than in cardiovascular tissues, highlighting its main function in the adrenal glands. Given that excessive aldosterone is linked to secondary hypertension and cardiovascular complications, the decreased activity of NR5A1 could contribute to CVDs [62]. In addition, another study showed a case report of a female fetus with a 46 XY karyotype who had congenital heart defects (CHD). Genetic testing revealed a microdeletion in the *NR5A1* gene, which was linked to a unique phenotype that included both DSD and CHD [63].

### ZFPM2 (zinc finger protein, FOG family member 2)

*ZFPM2*, also known as *FOG2*, is a multitype zinc finger cofactor that binds to and regulates the transcriptional activity of *GATA4*, a member of the GATA family of transcription factors [64]. Tevosian et al. (2002) demonstrated that *GATA4* and its co-factor, *FOG2*, are essential for normal gonadal development in mammals [16]. In mouse fetuses that lack *Fog2* or possess a mutated *Gata4* that interferes with their interaction, *Sry* expression is significantly decreased, resulting in atypical testis development [16]. Furthermore, while genes associated with ovarian development are differently activated, key genes essential for Sertoli and Leydig cell function are not expressed. This highlights the crucial roles of *GATA4* and *FOG2* in guiding the testis differentiation pathway [16]. Moreover, Crispino et al. (2001) established that GATA4’s role in heart development depends on its interaction with FOG2 and potentially other FOG proteins, as shown by the generation of mice with a *Gata4* mutation that disrupts this interaction [65]. The study showed that these mice die around embryonic day 12.5 and exhibit severe heart defects, including issues with semilunar valves and a double-outlet right ventricle, highlighting the importance of the GATA4 interaction in heart morphogenesis and coronary vascular development [65].

## Signaling pathways

### SMAD3 (SMAD family member 3)

*SMAD3* influences *TGF-β* and activin intracellular signaling pathways, which are important pathways for ovarian formation and regulation [66, 67]. *Smad3* deficiency in mice leads to reduced fertility due to impaired folliculogenesis, which affects follicle growth, atresia, and differentiation. *Smad3*^−/−^ mice exhibit slower follicle growth, increased atresia, and altered follicular differentiation [68]. These changes include modifications in hormone levels and receptor expression, suggesting that SMAD3 interacts with FSH signaling in the ovary, thereby influencing follicle development and ovulation [68]. Furthermore, Tsai et al. (2009) found that TGF-β/SMAD3 enhances vascular smooth muscle cells (VMCs) proliferation rather than inhibiting it [69]. This occurs through a mechanism involving the phosphorylation and nuclear export of the cyclin-dependent kinase inhibitor p27 [69]. Elevated SMAD3 levels in response to injury promote VSMC proliferation and contribute to intimal hyperplasia [69]. Another study investigated the role of SMAD3 in the vascular response to injury using *Smad3-*null mice, highlighting the protective role of SMAD3 in regulating VSMC cell growth and matrix remodeling following injury [70].

### WNT4 (Wnt family member 4)

*WNT4* is essential for ovarian differentiation and functions as one of the activators of the WNT signaling pathway, which is vital for ovarian differentiation in both humans and mice [71–73]. Recent findings involving women with symptoms similar to WNT4 deficiency, such as Mayer–Rokitansky–Küster–Hauser syndrome [74], and studies on *WNT4* gene mutations and Müllerian duct atypicality [75, 76] have confirmed the important role of WNT4 mutations in ovarian and female reproductive tract development. Additionally, increased levels of WNT2 and WNT4 activate the β-catenin/NF-κB signaling pathways, promoting cardiac fibrosis in fibroblasts through the collaboration of Fzd4/2 and LRP6 [77]. This mechanism is linked to negative outcomes in patients with acute MI, suggesting that targeting and inhibiting WNT2 and WNT4 systemically could help improve cardiac function after MI [77]. WNT4 has recently been further identified as a key driver of VSMC proliferation. Tsaousi et al. (2011) demonstrated that platelet-derived growth factor BB induced VSMC proliferation can be effectively reduced by specifically knocking down *WNT4* [78]. Furthermore, intimal thickening following carotid artery ligation was diminished in *Wnt4* heterozygous mice or when Wnt inhibitory factor 1 was introduced [78]. Similarly, inhibiting Wnt signaling by overexpressing *sFPR1* also reduced VSMC proliferation, both *in vitro* and *in vivo* [79]. This indicates that WNT4 plays a significant role in vascular remodeling, which is a key contributor to various CVDs.

### WNT5A (Wnt family member 5 A)

Dominant Robinow syndrome is linked to mutations in *WNT5A*, which support a noncanonical signaling model where a Wnt ligand communicates through a tyrosine kinase receptor. This finding highlights the role of the WNT5A/ROR2 pathway in the development of human craniofacial, skeletal, and genital structures [80]. Chromosome atypicality associated with the limb, skeletal, genital, and craniofacial characteristics of Robinow syndrome were identified, emphasizing the genetic factors involved [81]. Furthermore, phenotypes observed in *Wnt5a* null and *Ror2* null mice, including anterior-posterior axis shortening, facial dysmorphism, genital hypoplasia, and cardiac defects, mirror those seen in Robinow syndrome patients [82–85]. Wnt pathway plays a crucial role in myocardial remodeling [86–88]. Specifically, WNT5A has been shown to promote cardiomyocyte hypertrophy, whereas inhibiting this pathway can reduce hypertrophy [89] and the progression of heart failure [90, 91]. Recently, WNT5A has also been linked to the development of fibrosis in the neonatal heart following cryoinjury [92]. Abraityte et al. (2017) reported that WNT5A levels were elevated in the serum of heart failure patients and were linked to worsening disease severity [93]. In detail, elevated WNT5A increased IL-6 and TIMP-1 production in cardiac fibroblasts through ERK1/2 signaling but did not affect β-catenin levels. Blocking ERK1/2 reduced the Wnt5a-induced release of these inflammatory factors, which indicates that WNT5A contributes to inflammation and fibrosis in heart failure [93].

### MAP3K1 (mitogen-activated protein kinase kinase kinase 1)

Pearlman et al. (2010) demonstrated that mutations in *MAP3K1* cause 46 XY DSD. Their study revealed that mapping an autosomal sex-determining gene to chromosome 5 in two families with 46 XY DSD revealed a splice-acceptor mutation in *MAP3K1* that disrupts RNA splicing and affects downstream signaling pathways [94]. Additionally, Warr et al. (2011) [95] reported that *Map3k1*-deficient XY embryos on this genetic background showed no significant defects in testis determination, although minor atypicality, including an increase in gonadal length, were observed [95]. Minamino et al. (2002) proved that MAPK/ERK kinase kinase-1 (MEKK1/MAP3K1) has an important impact on cardiac hypertrophy induced by Gαq signaling in mice [96]. The study showed endogenous MEKK1 activation by Gαq leads to increased cardiac mass and myocyte size, with its disruption improving ventricular function and preventing hypertrophy-related changes, suggesting that MEKK1 is a key mediator of Gαq-induced cardiac hypertrophy and a promising target for heart disease treatments [96].

### SEMA3A (semaphorin 3 A)

*SEMA3A* mutations are linked to hypogonadotropic hypogonadism by affecting signaling pathways that regulate gonadotropin-releasing hormone (GnRH) neurons, leading to impaired sexual development and reproductive function [97]. Furthermore, the loss of function of *SEMA3A* results in phenotypes related to Kallmann syndrome [98, 99]. In a recent study, a novel missense variant in the *SEMA3A* gene was identified in a Chinese family with Kallmann syndrome, causing a significant reduction in SEMA3A protein expression and impaired function in GnRH neurons [100]. Other studies have reported that GnRH neuronal migration defects, atypical olfactory bulb development, and hypogonadism appear in *Sema3a* knockout mice [101]. Li et al. (2023) showed that SEMA3A is upregulated in microvascular endothelial cells under chronic pressure overload, impairing angiogenesis and leading to microvascular rarefaction in heart disease [102]. This effect is mediated by Sema3A-containing extracellular vesicles that disrupt angiogenic responses by competing with vascular endothelial growth factor A for binding to neuropilin-1 [102]. Furthermore, van Gils et al. (2013) demonstrated that SEMA3A is dysregulated under pro-atherosclerotic conditions, contributing to endothelial dysfunction and vascular remodeling [103].

### IL17RD (interleukin 17 receptor D)

Miraoui et al. (2013) demonstrated that mutations in *IL17RD* are strongly associated with Kallmann syndrome [104]. The study found that mutations are linked to absent puberty and are found in both heterozygous and homozygous forms in individuals with Kallmann syndrome. These mutations frequently occur alongside other genetic variants in individuals with Kallmann syndrome, indicating a role in the oligogenic basis of the syndrome [104]. Additionally, IL17RD is closely associated with and colocalizes with IL-17R, playing a crucial role in mediating IL-17 signaling. An *IL17RD* mutant lacking the intracellular domain can dominantly suppress IL17R-mediated signaling [105]. Low serum levels of IL-17 are linked to an increased risk of death and recurrent MI, as shown by evidence from the Fast-MI study, which involved 981 patients [106]. This study also found that low IL-17 levels, combined with high soluble VCAM-1 levels, are associated with an especially high risk of adverse cardiovascular outcomes [106].

## Hormonal regulation

### POR (cytochrome p450 oxidoreductase)

P450 oxidoreductase deficiency is a rare condition in humans that leads to a unique form of congenital adrenal hyperplasia [107]. This deficiency often results in partial and combined enzymatic adrenal dysfunction, associated with DSD in 46 XX and 46 XY individuals [108]. It is frequently linked to skeletal atypicality such as Antley-Bixler syndrome [109–111]. Moreover, Lopez et al. (2022) reported that deletion of POR in endothelial cells causes significant cardiac remodeling under basal conditions [112]. This has demonstrated the functional consequences of an endothelial-specific inducible *Por* knockout (*ecPor*^−/−^) for the heart. *EcPor*^−/−^ male mice show an increased ratio of heart mass to body mass, indicating that the deletion of POR in endothelial cells may lead to cardiac remodeling and potentially hypertrophy [112]. Likewise, an increased diameter of cardiac myocytes has been observed, highlighting the relationship between POR and cardiac development [112].

### CYP11A1 (cytochrome P450 family 11 subfamily A member 1)

Hiort et al. (2005) reported a 46 XY patient with a homozygous mutation in the *CYP11A1* gene, disrupting the P450scc enzyme, who was born with complete sex reversal and severe adrenal insufficiency, yet survived despite minimal steroid production [113]. Another study further revealed significantly reduced or absent steroid production across all pathways and further genetic analysis identified a homozygous single nucleotide deletion in the *CYP11A1* gene, resulting in a premature stop codon and predicting a nonfunctional P450scc enzyme [113]. In addition, Kolli et al. (2018) reported *CYP11A1* variant p.E314K impairs the stability of the P450scc enzyme and studied this variant in four patients with primary adrenal insufficiency [114]. In detail, the study showed that all patients were compound heterozygous for the *p.E314K* variant and another known pathogenic variant, resulting in reduced stability and function of the P450scc enzyme and causing partial adrenal and gonadal dysfunction [114]. Moreover, CYP11A1 plays an important role in the metabolism of cholesterol and vitamin D, both of which are linked to CVD [115, 116]. Gao et al. (2020) revealed a link between polymorphisms in *CYP3A4* and *CYP11A1* and the risk of ischemic stroke in the Chinese population [117]. The sex-stratified study found that the *CYP11A1* rs12912592 polymorphism influenced ischemic stroke risk in males but not in females, suggesting a gender-specific difference in this risk association [117].

### CYP11B1 (cytochrome P450 family 11 subfamily B member 1)

Mutations in *CYP11B1* cause 11β-hydroxylase deficiency (11βOHD) related to congenital adrenal hyperplasia [118, 119]. This condition is characterized by low levels of plasma cortisol, along with increased concentrations of 11-deoxycortisol, 11-deoxycorticosterone, and androgens. Hypertension occurs in roughly two-thirds of individuals with elevated 11-deoxycorticosterone and its metabolites, often appearing early in life [119–121]. In general, mutations in the *CYP11B1* gene are the second most prevalent cause of congenital adrenal hyperplasia, representing 0.2% to 8% of all cases [122–124]. In females, these mutations typically result in masculinized external genitalia, while males may develop isosexual precocious puberty. All individuals with this condition show accelerated growth and early closure of the epiphyses, leading to a shorter stature [125, 126]. Furthermore, Huang et al. (2022) investigated the impact of *CYP11B1 *gene mutations on CAD risk in the Chinese Han population [127]. The study reported that specific variants (rs4534, rs6410, rs5283) were significantly associated with CAD risk, depending on age and gender, and could also influence diabetes and hypertension risk among CAD patients. Additionally, a specific *CYP11B1* haplotype was found to reduce CAD susceptibility [127]. Another study demonstrated the genetic association of steroid hormones linked to *CYP11B1* and the implications for sexual dimorphism in CAD [128].

#### CYP11B2 (cytochrome P450 family 11 subfamily B member 2)

*CYP11B2* encodes aldosterone synthase, an enzyme essential for converting 11-deoxycorticosterone to corticosterone and 18-hydroxycorticosterone to aldosterone. Mutations in *CYP11B2* can impair this process, causing conditions like hyponatremia, hyperkalemia, and failure to thrive [129]. The study found that certain genetic variants in the *CYP11B2* gene are more common in patients with hyperaldosteronism, suggesting these variants contribute to hyperaldosteronism susceptibility [130]. Moreover, polymorphic variations at the *CYP11B2* locus are linked to CVD due to a single nucleotide polymorphism, which results in the substitution of an arginine residue with lysine [131, 132]. White et al. (1999) demonstrated that the polymorphism shows varying correlations with aldosterone secretion and blood pressure [133]. Furthermore, it has been associated with increased left ventricular size and reduced baroreflex sensitivity in healthy individuals, both of which are linked to cardiovascular risk. Additionally, the study confirmed that this polymorphism is related to an increased risk of MI in men with high-risk dyslipidemia [133].

### CYP17A1 (cytochrome P450 family 17 subfamily A member 1)

**Fig. 4:**
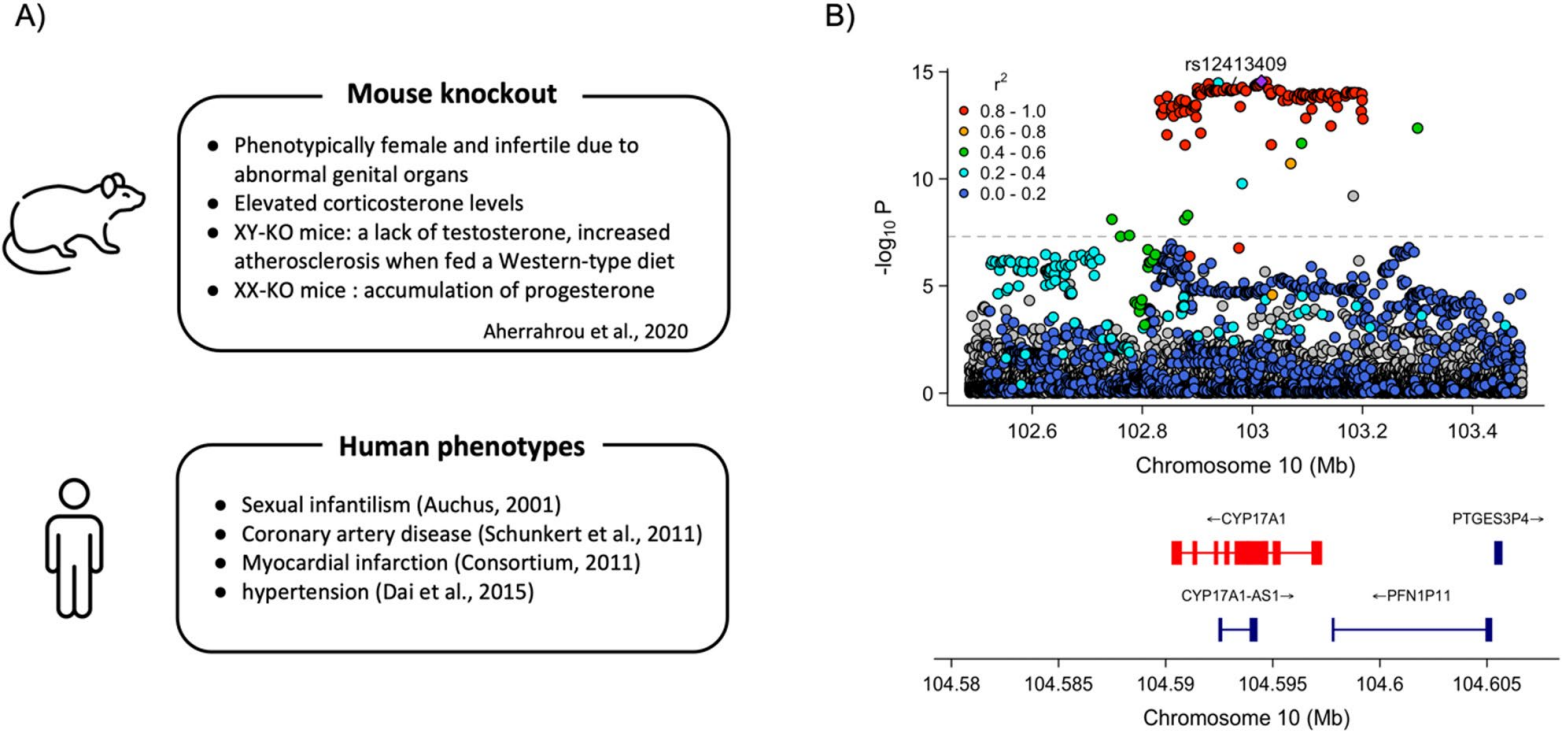
Comparative analysis of *Cyp17a1* in mouse knockout models and human phenotypes. **(A)** The figure depicts the results from mouse knockout studies and related human phenotypes, highlighting the essential role of the *CYP17A1* gene. **(B)** The figure shows a locus zoom plot for *CYP17A1*, indicating a significant association at the rs12413409 with -log10 P-values. The lower panel illustrates the gene structure and nearby genomic regions. The chromosome locations of the two panels are distinct to enhance clarity

*CYP17A1* gene encodes the enzyme 17α-hydroxylase/17,20-lyase, essential for the production of glucocorticoids and sex steroids, and is expressed in both the adrenal glands and gonads [134]. The 17α-hydroxylase activity is necessary for cortisol synthesis, while the 17,20-lyase activity is essential for the production of sex steroids [135]. Auchus (2001) first described 17-hydroxylase deficiency in patients with lack of sexual development and hypertension [136]. Auchus (2017) further detailed the genetic and pharmacologic aspects of these deficiencies, emphasizing their impact on steroid hormone production [137]. CYP17A1 is crucial for the biosynthesis of steroid hormones that regulate blood pressure, inflammation, and lipid metabolism and hormonal imbalances due to CYP17A1 deficiencies can lead to hypertension [138], CAD [139], and MI [140]. Additionally, SNP rs77787671 has been identified as a significant CAD risk locus, showing a strong genetic association with the phenotype [141, 142]. To further validate its role, we conducted knockout studies in mice, demonstrating that deficiencies in *Cyp17a1* result in phenotypic effects such as being phenotypically female with atypical genital organs, leading to infertility [143]. Additionally, we observed elevated corticosterone levels in knockout mice. Specifically, XY-KO mice show low levels of testosterone, while XX-KO mice exhibit increased atherosclerosis when fed a Western-type diet, along with elevated levels of progesterone [143] (Fig. 4).

### CYP19A1 (cytochrome P450 family 19 subfamily A member 1)

Mutations in the *CYP19A1* gene cause aromatase deficiency (AD), a rare autosomal recessive disorder that results in impaired conversion of androgens to estrogens [144]. Clinical features of AD include genital ambiguity at birth, primary amenorrhea, high levels of androgens with very low estrogen levels, and inadequate breast development during puberty in females [145–148]. Mazen et al. (2018) reported a case of AD in a 21-year-old Egyptian male with a 46 XX karyotype, who presented with a hypoplastic scrotum, a penis-like phallus, and the absence of testes [149]. The study further confirmed that a homozygous splice site mutation in the 46 XX karyotype is associated with aromatase deficiency [149]. Additionally, Peter et al. (2005) reported that polymorphisms in *CYP19A1* were linked to diastolic blood pressure in women [150]. Ziv-Gal et al. (2012) showed a linkage between genetic polymorphisms in the *CYP19A1* gene and the risk of hypertension among midlife women [151]. Another study further demonstrated that CAD risk loci are associated with variations in the *CYP19A1* gene and circulating sex hormone levels in the Chinese population [152].

### CYP21A2 (cytochrome P450 family 21 subfamily A member 2)

*CYP21A2* mutations cause congenital adrenal hyperplasia, resulting in 21-hydroxylase deficiency. This disrupts the synthesis of glucocorticoids and mineralocorticoids, leading to an excess of adrenal androgens and hormonal imbalance [153, 154]. Loss of CYP21A2 activity in the fetus can lead to excessive adrenal androgen production, which may not be regulated by the fetal-placental unit [155, 156]. Another study demonstrated common mutations in the *CYP21A2* gene and found strong genotype-phenotype correlations for congenital adrenal hyperplasia in the Turkish population [157]. It has been found that common genetic variants of *CYP21A2* are linked to changes in levels of hormone circulation. Specifically, the polymorphism in *CYP21A2* is linked to higher baseline aldosterone levels [158]. Changes in aldosterone levels could impact conditions such as resistant hypertension [159] and congestive heart failure [160].

### LEP (Leptin)

Leptin deficiency is associated with hypogonadotropic hypogonadism and can cause reproductive dysfunction through both central leptin resistance and direct effects on the gonads [161]. Clinical evidence shows that leptin gene mutations leading to complete leptin deficiency result in severe obesity, hyperphagia, insulin resistance, and significant neuroendocrine disturbances, including hypogonadism [162, 163]. Moreover, Leptin is related to the promotion of proliferation, differentiation, and functional activation of hematopoietic and embryonic cells, thereby facilitating myocyte growth [164, 165]. Several studies also indicate a direct connection between obesity-induced cardiac hypertrophy [166, 167] and heart failure [168]. Furthermore, blocking leptin receptors in rats with MI has been shown to cause hypertrophy and hemodynamic dysfunction [169]. Additionally, long-term leptin administration has been found to encourage the development of eccentric cardiac hypertrophy and eccentric left ventricular dilatation in rats [170].

## Developmental regulation

### CFTR (CF transmembrane conductance regulator)

*CFTR* gene mutations are linked to congenital absence of the uterus and vagina, a condition that occurs in about 1 in 5,000 females [171, 172]. Additionally, *CFTR* mutations are known to cause congenital bilateral absence of the vas deferens, and it occurs due to disruptions in the Wolffian ducts that develop into the male internal genitalia [172]. CFTR has been linked to cardiovascular health. Several research showed that CFTR dysfunction can lead to significant cardiovascular issues. For instance, Vizzardi et al. (2019) found that adults with cystic fibrosis, even those without common risk factors, showed problems in both macro- and microvascular function [173]. In another study, Baño-Rodrigo et al. (2012) discovered that adolescents with mild cystic fibrosis had right ventricular dysfunction, suggesting that heart problems can occur even in less severe cases [174]. Furthermore, Bright-Thomas and Webb (2002) demonstrated that chronic respiratory issues related to cystic fibrosis increase the workload on the heart, which could lead to complications like right ventricular hypertrophy [175]. Additionally, other studies have reported that CFTR dysfunction and the progression of cystic fibrosis lung disease affect heart function [176–178].

### HOXA13 (homeobox A13)

*HOXA13* shows a conserved pattern between mice and humans and is expressed in the upper vagina [179]. Mice with a targeted deficiency in the HoxD complex showed small digit primordia, a disorganized cartilage pattern and impaired skeletal mass [180]. These changes resemble the defects observed in human synpolydactyly, caused by mutations in the *HOXD13* gene [181–183]. Deletion in *Hoxa13* showed Hypodactyly and the rare surviving homozygotes of both sexes are infertile in mice [184–186]. The absence of HOXA13 function also affects placental endothelial cell morphology, resulting in compromised vessel wall integrity, edema in the embryonic blood vessels, and mid-gestational lethality [187–189]. This placental insufficiency has significant consequences for the development of fetal organs, particularly the heart, pancreas, lungs, and brain [190]. It accounts for about 60% of fetal growth restriction (FGR) cases in normally formed fetuses [191]. FGR is a significant predictor of health issues later in life. For instance, term neonates with low birth weight (less than 5.5 lbs) have an increased risk of mortality from CAD and a higher likelihood of developing diabetes and hypertension in adulthood [192–194].

### KLHL10 (kelch like family member 10)

*KLHL10* encodes a protein that is evolutionarily conserved and specifically expressed in spermatids [195]. Patients with oligozoospermia due to *KLHL10* mutations have been reported to have impaired homodimerization [196]. Furthermore, Cannarella et al. (2021) found a novel variant in patients with oligozoospermia and one with nonobstructive azoospermia, which is linked to male infertility [197]. In addition, Pan et al. (2023) revealed novel genetic alterations in *KLHL10* from patients with tetralogy of Fallot [198]. This association suggests that KLHL10 may play a role in CHD, which could affect the heart’s outflow tract [199–201].

### Challenges and future research directions

In terms of genetics, GWAS have discovered more than 40,000 associations between specific SNPs and traits from > 45,000 individual GWAS in ∼6,000 publications in the last decade [202]. However, interestingly very few of these trait-associated SNPs are located on the X or Y chromosome. This systematic neglect of sex chromosomes in analysis poses significant challenges, especially for understanding DSD, where sex chromosome variants play a crucial role, or CVD, where sex differences influence outcomes. This oversight is driven by several factors, as discussed by Wise et al. (2013) [203]. Early genotyping platforms included few markers for the X chromosome. Although the coverage has improved over time, it remains inferior to that of autosomes. The X chromosome’s lower gene density and differences in minor-allele frequency further complicate statistical power and association detection. Genotyping accuracy issues and the need for specialized analytical approaches due to sex-specific inheritance patterns prevent researchers from including X chromosome data. The Y chromosome faces similar neglect due to its structural complexity, lower gene density, high error rates in genotyping, and population-specific variation, as highlighted by Skaletsky et al. (2003) [204]. Some Y-SNP associations have been reported by targeted investigations, for example, in CAD [205]. However, it remains unclear whether the lack of genetic associations when it comes to X and Y chromosomes is because of true biological significance or if it is due to current methodological limitations.

Heterogeneity in genetic research poses another significant challenge, complicating the identification of causal variants and understanding complex diseases. This complexity can particularly challenge research on DSD and CVD because these conditions involve intricate genetic and phenotypic variations, and their pathology is highly variable. McClellan and King (2010) emphasize that individual mutations collectively play a substantial role in causing complex illnesses [206]. Different mutations in the same gene or different genes in related pathways can lead to the same disorder. This heterogeneity means that traditional large-scale associations or case-control studies often fail to resolve causality. Therefore, understanding and characterizing genetic heterogeneity is crucial, as the insights gained are essential for advancing therapeutic approaches [207, 208] and enhancing predictive accuracy [209].

Although we have evidence from a literature review of studies conducted using animals and humans that some DSD genes are linked to CVD, it is plausible that our current approach may underestimate the true biological relevance, as both DSD and CVD phenotypes can manifest many levels downstream of initial genetic perturbations, involving intricate metabolic, signaling, or gene regulatory networks. In addition, while not all individuals with 46 XY DSD will experience CVD, specific genetic factors can elevate the risk. Both the presentation of DSD and any associated CVD can vary significantly; therefore, genetic testing and multidisciplinary care are essential for accurate diagnosis and effective management of CVD in 46XY DSD individuals.

In this study, the genetic intersection between 46 XY DSD and CVD highlights the complexity of human development and disease. By exploring shared genetic pathways, we can gain valuable critical insights into both conditions. Additionally, understanding genetic overlaps could lead to a better comprehension of the long-term health risks for individuals with 46 XY DSD. Identifying genetic risk factors and understanding their roles in both conditions could potentially lead to personalized treatment strategies that account for individual genetic profiles.

## Supplementary Information

Supplementary Material 1

## Data Availability

No datasets were generated or analysed during the current study.

## Abbreviations

AD: Aromatase deficiency
CAD: Coronary artery disease
CVD: Cardiovascular diseases
CFTR: Cystic fibrosis transmembrane conductance regulator
CHD: Congenital heart disease
CYP: Cytochrome P450
CYP11A1: Cytochrome P450 family 11 subfamily A member 1
CYP11B1: Cytochrome P450 family 11 subfamily B member 1
CYP11B2: Cytochrome P450 family 11 subfamily B member 2
CYP17A1: Cytochrome P450 family 17 subfamily A member 1
CYP19A1: Cytochrome P450 Ffamily 19 subfamily A member 1
CYP21A2: Cytochrome P450 family 21 subfamily A member 2
DSD: Disorders of sex development
EV: Extracellular vesicles
FGR: Fetal growth restriction
FOG2: Friend of GATA Family 2
FSH: Follicle-stimulating hormone
GATA4: GATA binding protein 4
GnRH: Gonadotropin-releasing hormone
HOXA13: Homeobox A13
IL-17: Interleukin 17
IL17RD: Interleukin 17 receptor D
KLHL10: Kelch-like family member 10
KO: Knockout
LEP: Leptin
MAP3K1: Mitogen-activated protein kinase kinase kinase 1 (also known as MEKK1)
MI: Myocardial infarction
MWS: Mowat–wilson syndrome
NR5A1: Nuclear receptor subfamily 5 group A member 1
POR: Cytochrome P450 Oxidoreductase
SEMA3A: Semaphorin 3 A
SIP1: Smad-interacting protein 1
SMAD3: SMAD family member 3
SNP: Single nucleotide polymorphism
SOX9: SRY-box transcription factor 9
TCF21: Transcription factor 21
TGF-β: Transforming growth factor beta
VCAM-1: Vascular cell adhesion molecule 1
VSMC: Vascular smooth muscle cells
WT1: Wilms tumor 1
WNT4: Wnt family member 4
WNT5A: Wnt family member 5 A
ZEB2: Zinc finger E-box binding homeobox 2
ZFPM2: Zinc finger protein, FOG family member 2
